# Crowd-Sourced Mapping of New Feature Layer for High-Definition Map

**DOI:** 10.3390/s18124172

**Published:** 2018-11-28

**Authors:** Chansoo Kim, Sungjin Cho, Myoungho Sunwoo, Kichun Jo

**Affiliations:** 1 Department of Automotive Engineering, Hanyang University, 222 Wangsimni-ro, Seongdong-gu, Seoul 04763, Korea; chansoo7857@gmail.com (C.K.); sungjincho215@gmail.com (S.C.); msunwoo@hanyang.ac.kr (M.S.); 2 Department of Smart Vehicle Engineering, Konkuk University, Seoul 05029, Korea

**Keywords:** crowd-sourced mapping, HD map, new feature layer, map cloud, intelligent driving

## Abstract

A High-Definition map (HD map) is a precise and detailed map composed of various landmark feature layers. The HD map is a core technology that facilitates the essential functions of intelligent vehicles. Recently, it has come to be required for the HD map to continuously add new feature layers in order to increase the performances of intelligent vehicles in more complicated environments. However, it is difficult to generate a new feature layer for the HD map, because the conventional method of generating the HD map based on several professional mapping cars has high costs in terms of time and money due to the need to re-drive on all of the public roads. In order to reduce these costs, we propose a crowd-sourced mapping process of the new feature layer for the HD map. This process is composed of two steps. First, new features in the environments are acquired from multiple intelligent vehicles. The acquired new features build each new feature layer in each intelligent vehicle using the HD map-based GraphSLAM approach, and these new feature layers are conveyed to a map cloud through a mobile network system. Next, the crowd-sourced new feature layers are integrated into a new feature layer in a map cloud. In the simulation, the performance of the crowd-sourced process is then analyzed and evaluated. Experiments in real driving environments confirm the results of the simulation.

## 1. Introduction

Recently, most automobile companies have begun the development of intelligent vehicles such as vehicles with Advanced Driver Assistance Systems (ADAS), partially autonomous vehicles, and fully autonomous vehicles. These newly developed intelligent vehicles must satisfy various driving requirements such as safety, comfort, and fuel efficiency.

The foundation of an intelligent vehicle is its ability to understand the environment around it [1,2]. For this purpose, a variety of internal sensors such as ultrasonic sensors, RAdio Detection And Ranging (RADAR) sensors, LIght Detection And Ranging (LIDAR) sensors, cameras, and Global Navigation Satellite Systems (GNSS) are installed in intelligent vehicles. However, there are two limitations to understanding the environment which depends only on these internal sensors: the absence of integrity and the perception range. First, the integrity of the information acquired from internal sensors is not guaranteed; the sensors are sometimes vulnerable to environmental noises and are limited by the fact that high-performance computing units cannot be used in vehicles due to their high price and the excessive electric consumption. These problems make it so that the sensors cannot guarantee the complete tracking of the sensing information and real-time performance. In addition, these sensors have limited perception ranges, and the sensors cannot measure target objects located outside of its Field of View (FoV) or target objects occluded by other obstacles.

In order to solve these problems, a High-Definition map (HD map) have been widely researched [3]. The HD map is a precise and detailed map that stores landmark features which are essential to intelligent driving. Though the composition of the HD map differs according to the map providers (HERE [4] and TomTom [5]) or the purpose of the map (localization [6,7,8,9,10], perception [11], and planning [12]), the HD map generally includes various landmark features (such as road geometry [13], road maneuver [14], lane [15,16], road surface marking [17], and traffic control units [18]).

The use of the HD map improves several functions of the intelligent vehicles: localization, perception, and planning. First, the matching relation between the HD map and the perception measurements improves the accuracy and reliability of vehicle localization without the need for high-cost sensors [6,7,8,9,10]. Next, when the position and heading of the vehicle are known precisely by the vehicle localization, the features in the HD map can be used as virtual sensors in the intelligent vehicle. For example, the traffic sign information in the HD map can be “sensed” by the intelligent vehicle without using the perception sensors. In addition, in order to accurately recognize continuously changing information such as the state of a traffic light in real-time, information such as the position and size of the traffic light can be transmitted to the recognition system through the HD map [11]. Finally, the HD map can be used to generate the local routes and global routes to destinations in the path planning of the intelligent vehicle [12].

In order to generate the HD map used in intelligent vehicles, professional mapping vehicles equipped with Mobile Mapping System (MMS) are mainly used through three processes [19]. First, the mapping vehicle equipped with high-performance position and perception sensors travels along target routes in order to acquire the mapping data (data acquisition). Next, the features acquired from the mapping vehicle are accumulated based on the trajectory of the vehicle on the map according to the types of the features (data accumulation). Finally, the features in the map are manually refined and confirmed (data confirmation).

The constructed features in the HD map for the intelligent vehicles are generally managed by the layer-based map management system, which has some advantages in the application for the intelligent vehicle. Editing of the HD map such as addition, deletion, or correction, is efficient because it is easy to visualize and access the map database. In addition, the network bandwidth can be saved by downloading only the required layer, when the map data are downloaded to the intelligent vehicles. Finally, it is easy to support old vehicles, because the formats of the layers required by the vehicles remain despite the addition of the new layers.

Recently, it has come to be required for the HD map to add new feature layers continuously as a solution for more complicated problems in intelligent vehicles, as the functional requirements for intelligent vehicles have increased. Unfortunately, the new feature layer mapping in the conventional method using mapping vehicles has several problems: costs, human resources, and latency. (1) Since mapping vehicles must be re-driven on all roads to acquire new feature data, it requires substantial costs. (2) Many human resources are needed to confirm the newly acquired feature information. (3) Latency necessarily occurs in the addition of the new feature layers, because it takes lots of the times for few mapping vehicles to acquire all of the necessary data.

In order to solve the problems associated with new feature layer mapping based on several mapping vehicles, we propose a crowd-sourced mapping process of the new feature layer based on multiple intelligent vehicles. The main contribution of our paper is the generation of new feature layers in the accuracy level of the HD map using the existing HD map and crowd-sourced information without additional costs, human resources, or latency. Since the layers can be used in intelligent driving, it is essential for the new feature layers to be highly accurate.

For the new feature layers to be highly accurate, the fact that the crowd-sourced information acquired from intelligent vehicles is not as accurate as information acquired from mapping vehicles must be overcome. In order to overcome the inaccuracy of the information, the paper uses two concepts: matching with the HD map and crowd-sourced data.

• **Matching with the HD map:** By considering the matching between the sensor information and the existing features in the HD map, new feature layers are generated with increased precision.

• **Crowd-sourced data:** Combining many new feature layers acquired from many intelligent vehicles solves the accuracy problem that arises when using inaccurate sensors.

The results of the proposed algorithm were evaluated by simulations and confirmed by the experiments in the real driving environments.

In order to explain the mapping of the new feature layer based on two concepts, this paper is organized as follows. Section 2 presents a system architecture of the proposed system. Section 3 explains the new feature layer mapping in an individual intelligent vehicle. Section 4 describes the integration of the new feature layers acquired from multiple intelligent vehicles in a map cloud system, and Section 5 evaluates the proposed system through simulations. Section 6 explains the experimental results. Finally, the conclusion is presented in Section 7.

## 2. System Architecture

**Figure 1:**
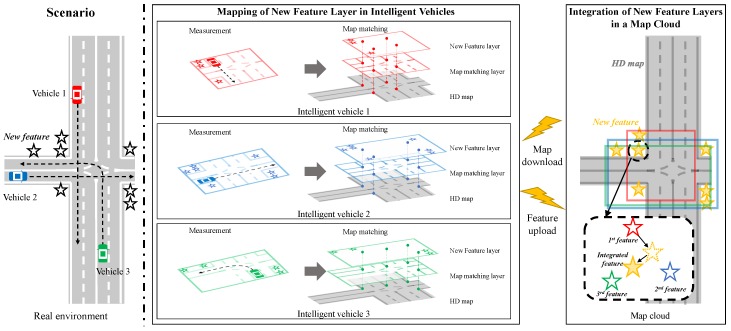
The crowd-sourced mapping process of the new feature layer with a map cloud system.

The crowd-sourced mapping process of the new feature layer for the HD map consists of two steps, as shown in Figure 1. The two steps are composed of (1) the mapping of the new feature layer in each intelligent vehicle and (2) the integration of the new feature layers in a map cloud. First, each intelligent vehicle acquires the vehicle speed, yaw rate, GNSS, and perception measurements (including new features and the map-matching features to match with the existing features in the HD map) to estimate the pose of the ego-vehicle precisely. The data can also be used as the mapping data to generate the new feature layer for the HD map. In order to perform the mapping process, an HD map-based incremental GraphSLAM algorithm is applied [20]. The algorithm accumulates the mapping data incrementally during driving on the roads. When the vehicle is stopped temporally or shut-down at the parking lot, the algorithm optimizes the acquired new features using the GraphSLAM algorithm. After the optimization, the new feature layer optimized in the vehicle is uploaded to the map cloud. Also, the algorithm is initialized and starts to re-accumulate the mapping data. Since the algorithm is based on the optimization method, the algorithm can estimate new features more precisely than filtering-based methods such as EKF-SLAM [21] and FastSLAM 2.0 [22]. In addition, since the GraphSLAM algorithm uses matching constraint between the map-matching features and the existing features in the HD map downloaded from the map cloud, the new features are estimated precisely. Each new feature layer constructed in each intelligent vehicle is uploaded to the map cloud through a mobile network system. Since the new feature layer is compressed from mapping data to some new features, the network bandwidth can be saved.

Next, multiple new feature layers conveyed by the multiple intelligent vehicles are combined into an integrated new feature layer in the map cloud. New features estimated by individual vehicles are associated with an integrated new feature through data association algorithms such as distance-based clustering. The map cloud uses a recursive least square (RLS) algorithm to update the integrated feature layer whenever individual new feature layers are uploaded from multiple vehicles [23].

## 3. Mapping of the New Feature Layer in an Intelligent Vehicle

### 3.1. New Feature Layer Mapping Without HD Map

**Figure 2:**
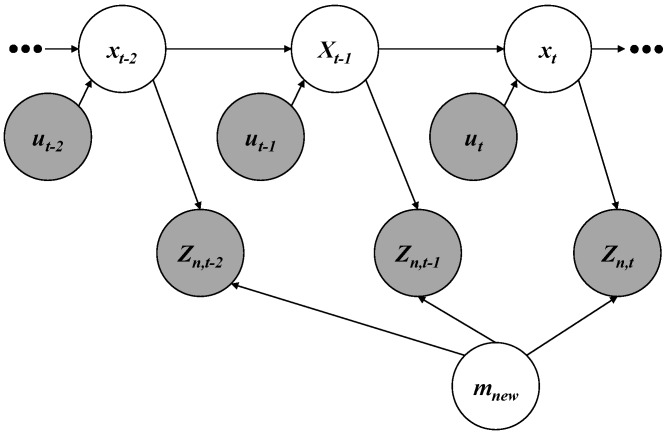
Graph representation for the new feature layer mapping.

In order to perform the mapping process in each intelligent vehicle, the GraphSLAM algorithm is used [20]. The algorithm is composed of three components: nodes, edges, and a solver. The nodes refer to the estimate states and measurements. As shown in the graph representation of Figure 2, the nodes are represented as circles. The edges mean the relative constraints between nodes. In Figure 2, the edges are represented as the arrows connecting two nodes. Finally, the solver solves the new feature mapping problem to estimate vehicle poses and new features using optimization algorithms such as the Gauss-Newton algorithm and the Levenberg-Marquardt algorithm.

#### 3.1.1. Node

While an intelligent vehicle is driving along the target trajectory that includes poses $x_{1:t}=\left\{x_{1},\cdots ,x_{t}\right\}$, the vehicle acquires motion information $u_{1:t}=\left\{u_{1},\cdots ,u_{t}\right\}$ and new feature measurements $z_{n,1:t}=\left\{z_{n,1},\cdots ,z_{n,t}\right\}$. As shown in Figure 2, the nodes are composed of vehicle poses $x_{1:t}$, motion information $u_{1:t}$, new feature measurements $z_{n,1:t}$, and the new feature layer $m_{new}=\left\{m_{n,1},m_{n,2},\cdots ,m_{n,N}\right\}$ which includes *N* new features. The poses $x_{1:t}$ can be represented as two-dimensional special Euclidean groups (SE(2)). The SE(2), represented as the heading angle and the position in a plane, contains the yaw angle and the relative distances to the east and the north from a specific reference point. The motion information $u_{1:t}$ is composed of the velocity and the yaw rate of the vehicle. The new feature layer $m_{new}$ in a global coordinate and new feature measurements $z_{n,1:t}$ in a vehicle coordinate can be parameterized in various forms such as spatial landmarks, occupancy grids, and point clouds.

The optimization method, which is the basis of the GraphSLAM algorithm, requires an initial point to estimate the states. The initial point is represented in a high-dimensional space composed up of poses $x_{1:t}$ and new features in the new feature layer $m_{new}$. In order to initialize the poses, Thrun et al. used motion information and the vehicle motion model [24]. However, this method may accumulate drift errors by biased noises of motion sensors over the times. Similarly, a Bayesian filtering-based approach such as the extended Kalman filter and the unscented Kalman filter which integrates motion information and GNSS information can cause errors in terms of the poses in the poor GNSS region. In order to overcome this problem, the optimal smoothing algorithm is applied for the global estimation of initial poses based on motion information and GNSS information [13,16]. The initial poses can initialize the new features in the new feature layer. All of the new feature measurements in vehicle coordinates can be converted to the global coordinate using the poses from where each measurement was acquired. In the global coordinate, new feature measurements adjacent to each other are associated as a new feature in the new feature layer. In this process, the associated new features become the initial new features.

#### 3.1.2. Edge

In the graph representation of Figure 2, an edge connecting two nodes is a constraint between two states. The edges are composed of the transition model $p(x_{t}|x_{t-1},u_{t}))$ and the new feature measurement model $p(z_{n,t}|x_{t},m_{new})$. The transition model is interpreted into a motion constraint between two consecutive poses. The pose $x_{t}$ at time *t* can be propagated from the pose $x_{t-1}$ at time t−1 using motion information $u_{t}$.

(1) $$x_{t}=g\left(x_{t-1},u_{t}\right)+\epsilon_{p,t}$$

(2) $$F_{p,t}=\epsilon_{p,t}^{T}\Omega_{p,t}\epsilon_{p,t}$$

The Equation (1) is the transition model with the error $\epsilon_{p,t}$. Since the transition model is uni-modal, the $\epsilon_{p,t}$ can be modelled as a Gaussian noise with zero means and information matrix $\Omega_{p,t}$. Therefore, the motion constraint generates the negative log likelihood $F_{p,t}$ as shown in Equation (2).

The new feature measurement model makes a new feature constraint where measurements of perception sensors are associated as a new feature in the new feature layer $m_{new}$. The new feature measurements $z_{n,t}$ at the pose $x_{t}$ can be modelled by the pose $x_{t}$ and the new feature layer $m_{new}$.

(3) $$z_{n,t}\approx h_{n}\left(x_{t},m_{new}\right)$$

(4) $$z_{n,t}^{i}=h_{n}\left(x_{t},m_{new,j}\right)+\epsilon_{n,i,t}$$

(5) $$F_{n,i,t}=\epsilon_{n,i,t}^{T}\Omega_{n,i,t}\epsilon_{n,i,t}$$

Equation (3) is the new feature measurement model which estimates the new feature. The measurement model Equation (3) is multi-modal, due to there being no correspondence with the measurements and the layer. The measurement model $h_{n}\left(x_{t},m_{new}\right)$ can be converted into a uni-modal measurement model $h_{n}\left(x_{t},m_{new,j}\right)$ where $j=c(x_{t},z_{n,t}^{i},m_{new})$ is the corresponding index between *i*-th new feature measurement $z_{n,t}^{i}$ and new feature layer $m_{new}$. In the uni-modal measurement model Equation (4), the new feature error $\epsilon_{n,i,t}$ can be modelled by Gaussian noise with zero mean and information matrix $\Omega_{n,i,t}$. Therefore, the new feature constraint generates the negative log likelihood $F_{n,i,t}$ in the Equation (5).

#### 3.1.3. Solver

A cost function for the optimization of the GraphSLAM algorithm is derived from the sum of the negative log likelihoods Equations (2) and (5).

(6) $$J=\sum_{t}\epsilon_{p,t}^{T}\Omega_{p,t}\epsilon_{p,t}+\sum_{i,t}\epsilon_{n,i,t}^{T}\Omega_{n,i,t}\epsilon_{n,i,t}$$

The unknown variables $x_{t}$ and $m_{new}$ can be estimated by minimizing the cost function Equation (6) based on the graph optimization [20,25]. While they estimate only poses and not map information [20], the proposed approach estimates all states including poses and map information.

### 3.2. HD Map-Based New Feature Layer Mapping

The new feature mapping algorithm explained in Section 3.1 considers motion information and new feature measurements without using the HD map. Using the additional information such as the HD map and the map-matching feature measurements, the new feature mapping algorithm can be improved because the map-matching constraints between the map-matching feature measurements and the existing features in the HD map can improve the estimation performance.

#### 3.2.1. Node

**Figure 3:**
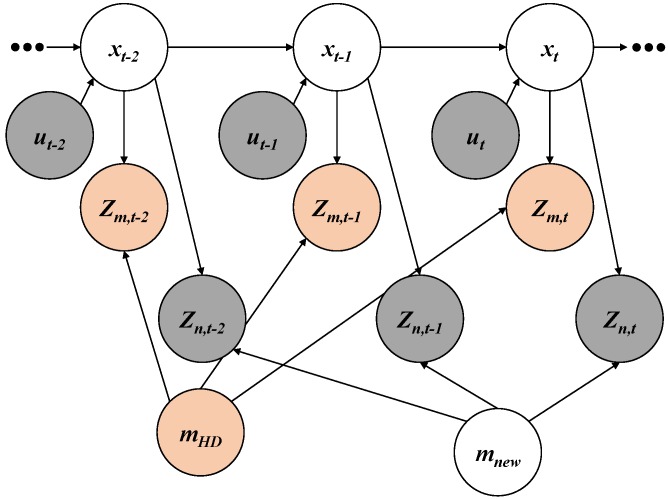
Graph representation for HD map-based new feature layer mapping.

As shown in Figure 3, the nodes of the HD map $m_{HD}$ and the map-matching feature measurements $z_{m,t}$ are added to the graph representation. In a similar manner to the new feature layer and the new feature measurements, the HD map and map-matching feature measurements can be parameterized into different forms such as landmarks, occupancy grids, and point clouds.

#### 3.2.2. Edge

A map-matching model between the existing features in the HD map and map-matching feature measurements is converted to a constraint based on a map-matching algorithm. Such considering of the matching relation can increase the accuracy of the poses and the new features, because more accurate poses can propagate more accurate new features. The map-matching feature measurement $z_{m,t}$ at time *t* can estimate the vehicle pose $x_{t}$ in consideration of the matching with the HD map $m_{HD}$.

(7) $$x_{t}\approx h_{m}\left(m_{HD},z_{m,t}\right)$$

Equation (7) is the map-matching feature-based pose estimation model. Since the model is a multi-modal model, it must be converted to a uni-modal model. In order to convert the uni-modal model, the initial pose ${\overset{˘}{x}}_{t}$ is used.

(8) $$x_{t}=h_{m}\left({\overset{˘}{x}}_{t},m_{HD},z_{m,t}\right)+\epsilon_{m,t}$$

(9) $$F_{m,t}=\epsilon_{m,t}^{T}\Omega_{m,t}\epsilon_{m,t}$$

Using the uni-modal model Equation (8), the map-matching error $\epsilon_{m,t}$ can be modelled by a Gaussian noise with zero mean and information matrix $\Omega_{m,t}$. Therefore, the map-matching based pose estimation constraint generates negative log likelihood $F_{m,t}$ in the Equation (9).

#### 3.2.3. Solver

A cost function for the optimization of the new feature layer mapping with HD map is derived from the sum of the negative log likelihoods Equations (2), (5), and (9).

(10) $$J^{\prime }=\sum_{t}\epsilon_{p,t}^{T}\Omega_{p,t}\epsilon_{p,t}+\sum_{i,t}\epsilon_{n,i,t}^{T}\Omega_{n,i,t}\epsilon_{n,i,t}+\sum_{t}\epsilon_{m,t}^{T}\Omega_{m,t}\epsilon_{m,t}$$

Since the map-matching constraints are used in the optimization, the unknown variable $x_{t}$ and $m_{new,j}$ can be estimated by minimizing the cost function Equation (10) more precisely than the cost function Equation (6).

## 4. Integration of New Feature Layers in a Map Cloud

The GraphSLAM algorithm generates a new feature layer on the driven roads whenever the vehicle is stopped temporally or at a parking lot. The generated new feature layer is uploaded to the map cloud through the mobile network system. This approach can reduce the network bandwidth because the transmitting data are compressed from the mapping data to the new features in the layer through the GraphSLAM algorithm.

Although the HD map is considered in the estimation of the vehicles, the crowd-sourced new feature layers uploaded from multiple intelligent vehicles may have errors in actual positions of the new features due to the inaccuracy of the low-cost sensors. In order to minimize the errors of new features, the RLS algorithm ([23]) generates an integrated new feature layer by combining the crowd-sourced new feature layers from multiple intelligent vehicles. Since the RLS algorithm recursively estimates the integrated layer whenever the crowd-sourced layers are entered, the algorithm is suitable for integrating the crowd-sourced layers into the integrated layer.

The values and covariances of integrated new features must be initialized to apply the RLS algorithm.

(11) $$\begin{array}{c} x_{0}=y_{0},\;first\;new\;feature\\ P_{0}=R_{0},\;noise\;covariance\;of\;perception\;sensor \end{array}$$

The *x* and the *P* are the state and the covariance of the feature in the integrated new feature layer, respectively. The *y* refers to the new features estimated in each vehicle. A firstly-uploaded new feature $y_{0}$ is used to initialize an integrated new feature. The initial covariance of the integrated feature is determined by the noise covariance *R* of the perception sensor equipped in the intelligent vehicle. For the purpose of merging the crowd-sourced layers into the integrated layer, each measured new feature is associated to the integrated feature using the distance-based clustering algorithm. The associated new feature updates the integrated feature using the RLS algorithm.

(12) $$\begin{array}{ccc} K_{k} & = & P_{k-1}H_{k}^{T}{\left(H_{k}P_{k-1}H_{k}^{T}+R_{k}\right)}^{-1}\\ x_{k} & = & x_{k-1}+K_{k}\left(y_{k}-H_{k}x_{k-1}\right)\\ P_{k} & = & \left(I-K_{k}H_{k}\right)P_{k-1}{\left(I-K_{k}H_{k}\right)}^{T}+K_{k}R_{k}K_{k}^{T} \end{array}$$

The Jacobian matrix of the measurement model *H* is an identity matrix because the measurement *y* and the estimated states *x* are the same. Entering futher measurements into the RLS algorithm reduces the covariance and makes the integrated map more accurate.

## 5. Simulation

A simulation analysis was performed in order to evaluate the crowd-sourced mapping process of the new feature layer with the HD map and to analyze the effects of the noises of commercial sensors on the new feature layer mapping. In the simulation, a beacon layer is generated based on the crowd-sourcing mapping with the HD map which has a lane layer only. The beacon can be modeled by single points which have two-dimensional position information. The reason why the beacon is selected for the new features is that it is easy to intuitively understand the process of the new feature mapping algorithm. Not only the beacon but also other parameterized data can be generated as the new feature layer. On the other hand, the lane in the HD map can be modeled by polylines which have consecutive points to represent lines. Since the lane information exists on most of the actual roads, it is selected as the HD map in the simulation. Various feature types as well as the lane can be used as the HD map.

**Figure 4:**
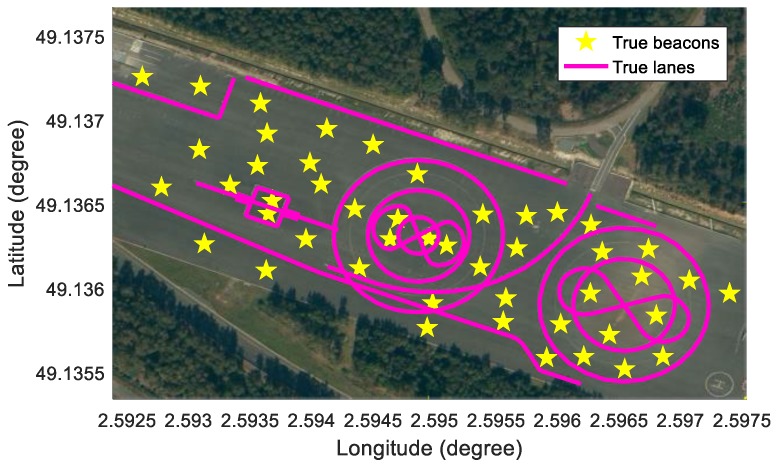
Test site for simulation: UTAC CREAM.

In order to perform the simulation, a test vehicle is equipped with a high-precision Global Navigation Satellite System/Inertial Measurement Unit (GNSS/IMU) with 0.01 m positioning errors, in-vehicle motion sensors, and a low-cost GNSS with 2.5 m positioning errors. To estimate new features, the mapping data consists of vehicle motion data (yaw rate and speed), a low-cost GNSS, and virtual noised lanes and beacons. The virtual lanes and beacons replace the actual features for the purpose of analyzing the effects of the sensor noises. The test site is UTAC CREAM in France, as shown in Figure 4.

### 5.1. Simulation of Sensors

**Figure 5:**
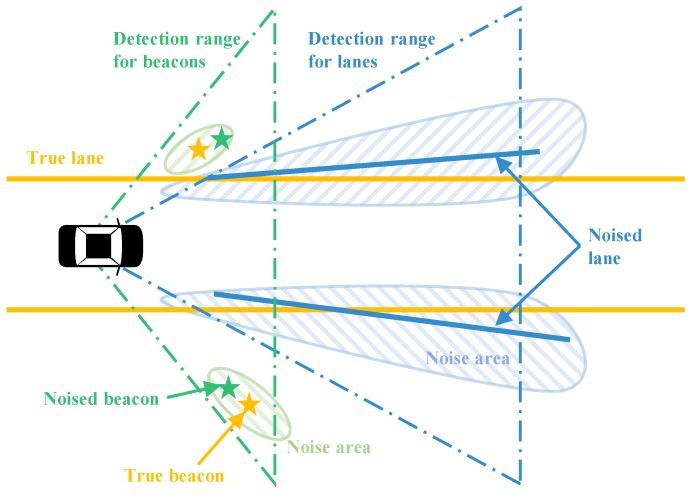
Concept of perception simulators for beacons and lanes.

The GNSS/IMU can measure the precise pose of a vehicle. The precise pose can be used to simulate the information which would otherwise be gathered by low-cost perception sensor information. First, a true beacon layer and a true lane layer need to be pre-defined in the WGS84 coordinates, as shown in Figure 4. The true beacon layer plays two roles in the simulation: (1) the source information to generate noised beacon measurements, and (2) the ground truth information to evaluate the beacons estimated by the proposed algorithm. On the other hand, the true lane layer also plays two roles in the simulation: (1) the source information to generate noised lane measurements, and (2) the existing feature layer in the HD map for matching with noised lane measurements. Second, when the test car is driven in the test site, the global positions of the beacons and the lanes within the detection range of the car can be inferred from the two true layers based on the precise GNSS information, as represented in Figure 5. Next, the beacons and the lanes in the WGS84 coordinates are converted in the vehicle coordinate so as to simulate the perception information. Finally, Gaussian white noises are added to the computed precise perception information to simulate the noised beacons and the noised lanes which can be measured by low-cost sensors. The perception sensor noises added to the sensor measurements are designed as functions of the noise level $l_{beacon}$ and $l_{lane}$ in Table 1. Basically, the two noise level parameters are initialized as 1.

To match between the simulated lanes (red) and the lanes in the HD map (blue), a generalized iterative closest point (GICP) algorithm is applied [26]. Since the GICP algorithm matches the source points with the target points, the simulated lanes and the lanes in the HD map are sampled to the points at intervals of 0.1 m. The matching relation between two lanes can infer the transformation matrix between the initial pose and the estimated pose.

### 5.2. Results of Simulation

The results of the new feature mapping algorithm in the simulation are evaluated using the Euclidean distance errors between the estimated beacons and the true beacons. In order to analyze the effects of the HD map in the algorithm, the simulation is split into two cases: new feature mapping without the HD map and new feature mapping with the HD map. Next, the results of map integration are computed so as to analyze the effects of the number of measurements.

**Figure 6:**
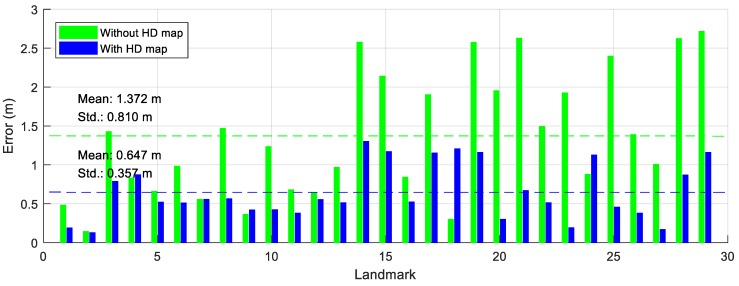
The errors of the new features.

The distance errors of the beacons are shown in Figure 6. The green bars in Figure 6 are the errors of the beacons generated by the new feature mapping algorithm without the HD map. The mean and the standard deviation of the errors without the HD map are 1.372 m and 0.810 m, respectively. The blue bars in Figure 6 represent the errors of the beacons generated by the new feature mapping algorithm with the HD map. The mean and the standard deviation of the errors with the HD map are 0.647 m and 0.357 m, respectively. The mean and the standard deviation of beacons considering the HD map are smaller than those of the beacons not considering the HD map, because matching information with the HD map is used to estimate the positions of the beacons. In other words, the beacons can be more precisely estimated when considering the HD map.

**Figure 7:**
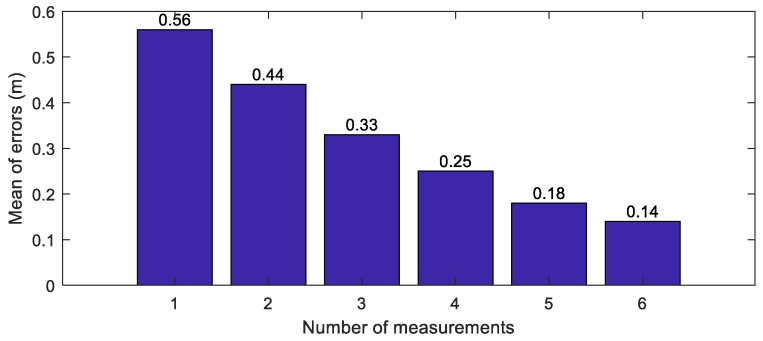
The errors of the integrated features.

For simulating the map integration, the crowd-sourced new feature layers update the integrated new feature layer using the RLS algorithm. In order to evaluate the accuracies of the beacons along the number of measurements, the Euclidean distance errors of the integrated beacons are analyzed. The means of the errors of the integrated beacons are represented along the number of the measurements, as shown in Figure 7. When the number of the measurements is one, the mean of errors of the beacons is 0.56 m. On the other hand, when the number of the measurements is 6, the mean of errors is 0.14 m. When more measurements are used, the means of the Euclidean distance errors further decrease.

### 5.3. Analysis of Sensor Noises

To simulate the errors associated with the low-cost perception sensors, the beacon simulator and lane simulator are used in the simulation. To analyze the effects of low-cost perception sensors, the means of the Euclidean distance errors of beacons are analyzed by changing the perception sensor noises.

**Figure 8:**
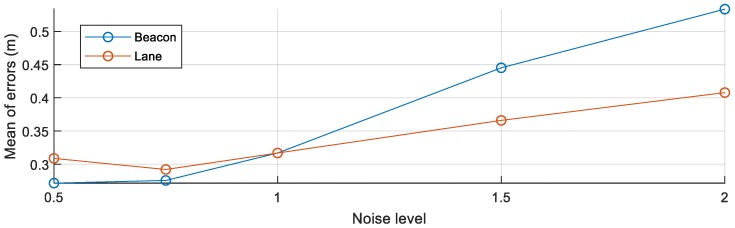
The mean of the errors of the integrated features along to the noises.

While the noise level *l* of a sensor is changed from 0.5 to 2, the noise level *l* of the other sensor is fixed to 1 for analyzing the effect of the noise of the target sensor. After the noise level of the target sensor is selected, beacons are estimated using the HD map-based new feature mapping algorithm and the RLS algorithm using crowd-sourced data. The beacons are evaluated by the true beacon map. The means of the errors of all beacons are shown in Figure 8. The blue line represents the change of the mean of the errors along the change of $l_{beacon}$ at $l_{lane}=1$, while the orange line represents the change of the mean of the errors along the change of $t_{lane}$ at $l_{beacon}=1$. When the noise level of the lane sensor increases, the means of errors of the beacons increase. Since the noised lanes have different shapes with the HD map, the rotation and translation matrices computed by the GICP algorithm are more incorrectly estimated along the more noised lanes. The most significant effect on the result is the noise of the beacon sensor. When the noise of the beacon sensor increases, the estimated beacons become inaccurate, because the noised information is directly combined with the estimated positions of the beacons.

## 6. Experiment

### 6.1. Experimental Environment

**Figure 9:**
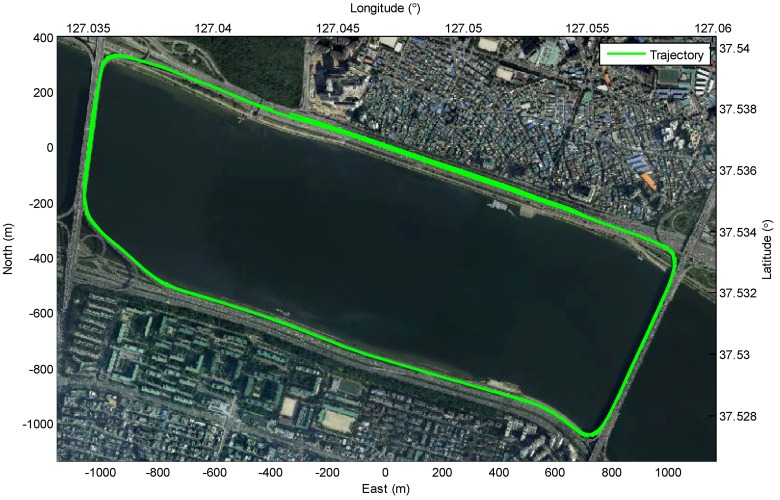
Test site for experiment: motorway road in Korea.

The experiments are evaluated in two ways: (1) crowd-sourced mapping of the new feature layer and (2) localization on the HD map with the generated new feature layer. A test site for the experiment is a motorway road in Korea, as shown in Figure 9. A streetlight layer is generated as new feature layer based on the crowd-sourced mapping process with the HD map which includes a lane layer. The reason why the streetlight is selected as the new feature is that the streetlights most frequently appear on the test site. Since the lanes which are basic elements of the road are on all of the public roads, the lanes are used as map-matching features. In order to generate the new feature layer, it is necessary to acquire the multiple mapping data from lots of intelligent vehicles. Since it is difficult to prepare lots of intelligent vehicles, the mapping data are replaced by data acquired from multiple drivings of a test vehicle (11 drivings). The generated new feature layer (the streetlight layer) is evaluated by comparing with the true streetlight layer which is generated by the conventional mapping process based on the high-precision sensors. On the other hand, in order to evaluate the effect of the new feature layer in the intelligent driving, the performance of the localization based on the HD map with the generated new feature layer was compared with the performance of the localization based on the HD map only. The performance of the localization is evaluated by the high-precision sensors.

In order to evaluate the algorithm in two ways such as mapping and localization, the test vehicle is equipped with a commercial camera (Mobileye EyeQ3), a LIDAR with 16 layers (Velodyne VLP-16), a high-precision GNSS/IMU (OXTS RT3002), a low-cost GNSS(U-blox EVK-6T), and in-vehicle motion sensors (a yaw rate sensor and a wheel speed sensor). The camera can detect the lane information at 15 FPS in the 40° horizontal view. The LIDAR with 16 layers can detect the point clouds reflected from the surrounding environment at 10 FPS in the 360° horizontal view. Although either the image processing approach [27] or deep learning approach [28] can be applied to detect the streetlights, the pole-like object detector based on the multi-layer LIDAR [29] is applied for convenience in the experiments. The high-precision GNSS/IMU and the low-cost GNSS have 0.01 m and 2.5 m position accuracy, respectively.

It is essential to generate two types of data: (1) the true streetlight layer to evaluate the mapping performance of the proposed algorithm and (2) the lane layer used as the HD map. In order to generate both units of data, the conventional mapping method is applied. The point clouds from the LIDAR are accumulated based on the vehicle pose measured by the high-precision GNSS/IMU. The true streetlight layer is extracted manually based on the shape of accumulated point clouds. The true streetlight layer is used to evaluate the mapping performance of the new feature layer generated by the proposed algorithm based on crowd-sourced mapping data. Similarly, the lane layer is extracted manually based on the intensity of accumulated point clouds. The lane layer used as the HD map increases the mapping performance of the new feature layer by matching with the lanes measured by the commercial camera.

### 6.2. Mapping of New Feature Layer

**Figure 10:**
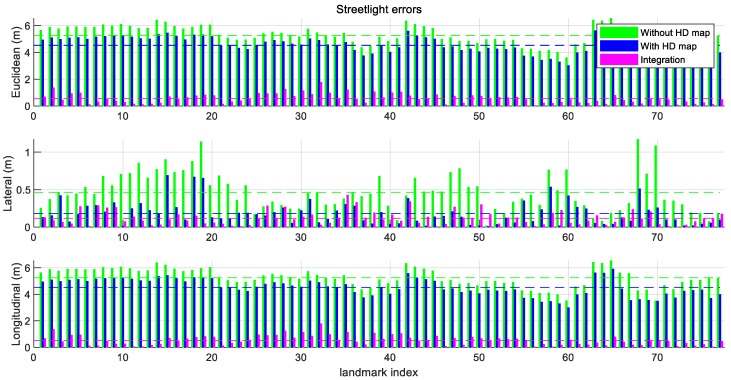
Errors of estimated streetlights. (top) the Euclidean error, (middle) the lateral error, and (bottom) the longitudinal error of the streetlights. The lateral errors are reduced based on the matching with the HD map. The longitudinal errors are reduced by considering the crowd-sourced data.

**Figure 11:**
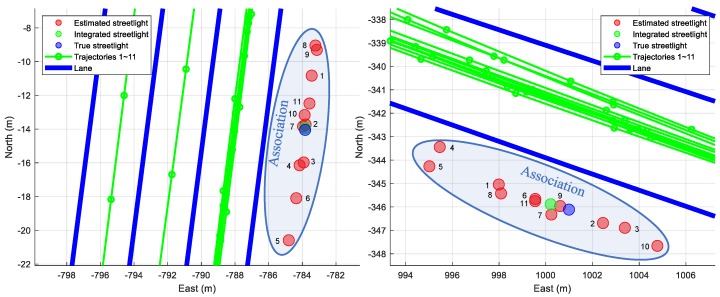
Results of the proposed algorithm in the specific streetlights. The integrated streetlight is updated recursively based on each indexing number beside each estimated streetlight. As a result, the integrated streetlight approximates the true streetlight.

To evaluate the results of both the individual new feature layer estimated by one mapping data and the integrated new feature layer estimated by multiple mapping data, the Euclidean distance errors of the estimated streetlights are shown in Figure 10. In contrast, only considering the Euclidean distance errors for the evaluation in the simulation, the graphs in Figure 10 include Euclidean distance errors, lateral distance errors, and longitudinal distance errors. The lateral errors and longitudinal errors between the ground truths of the streetlights and the estimated streetlights refer to the differences in the perpendicular direction and the parallel direction of the trajectory of the vehicle, respectively. The reason for using three types of errors is to reflect the properties of lanes as follows. As shown in Figure 11, the lanes in the HD map cannot determine the longitudinal position of the vehicle because lanes are continuously represented as similar lanes which are placed straight toward the longitudinal direction in the motorway condition. While the lateral position of the car can be estimated by matching information of these lanes, the longitudinal position cannot be estimated precisely in the same way. Therefore, when the lane matching is considered, the streetlights are more precise to the lateral direction than they are to the longitudinal direction. On the other hand, the longitudinal errors of new features estimated with the HD map are caused by noises of sensors such as wheel speed sensors and low-cost GNSS. Since each new feature estimated by each driving is spread to the longitudinal direction around the ground truth of the streetlight as shown in Figure 9, the new feature integrated by the RLS algorithm approximates the ground truth of the streetlight.

The green bars in Figure 10 are generated by the new feature layer mapping without the HD map in a single intelligent vehicle. The blue bars in Figure 10 show the errors of the streetlights estimated by the HD map-based new feature layer mapping in the single driving. The magenta bars are made by the map integration using crowd-sourced new feature layers. The means and the standard deviations of the errors from several conditions are shown in Table 2.

As shown in Table 2, consideration of the HD map leads to a substantial difference in the mean of the errors. The mean of lateral errors of streetlights is reduced to 0.1825 m due to the consideration of the lane matching. In contrast, the mean of the longitudinal errors of the streetlights with the HD map is 4.5220 m which is similar to the mean of the longitudinal errors without the HD map, which is 5.2445 m, because the lane matching rarely compensates for the longitudinal errors.

The RLS algorithm integrates with crowd-sourced new feature layers. The means and standard deviations of Euclidean, lateral, and longitudinal errors based on the map integration were smaller than those not based on the map integration. The longitudinal errors especially can be reduced dramatically. The experiment integrating motion, GNSS, perceptions, and the HD map based on the crowd-sourced data resulted in the mean errors 0.5458 m in the Euclidean distance. Therefore, the experimental results demonstrate that streetlights are more precisely generated through matching with the HD map and crowd-sourced data.

### 6.3. Localization in HD Map With New Feature Layer

To evaluate the effect of the existence of the new feature layer in the localization of the intelligent vehicle, the localization algorithm is performed under two conditions: (1) the HD map without the new feature layer and (2) the HD map with the new feature layer. The test vehicle uses in-vehicle motion sensors, the low-cost GNSS, the camera sensor to detect the lanes, and the LIDAR sensor to detect the streetlights. The high-precision GNSS/IMU is used as the ground truth information of the vehicle pose.

To perform the localization, the GraphSLAM approach is applied. Under both of the conditions, the lane matching is considered in the manner explained in the Equation (8). The approach considers motion information, GNSS information, and lane matching information similarly with the general lane based localization algorithm [30]. Under the condition in the HD map with the new feature layer, the streetlight matching is additionally considered in the similar manner explained in the Equation (4). While the $m_{new,j}$ is used as a variable in the mapping process, $m_{new,j}$ is used as a fixed value in the localization process.

As shown in Table 3, the lateral Root Mean Square Errors (RMSEs) without the new feature layer 0.2214 m and with the new feature layer 0.2077 m are similar to each other, the reason being that matching with the lanes compensates for the lateral positioning errors. However, the longitudinal RMSE with the new feature layer 0.6261 m is smaller than the RMSE without the new feature layer 5.4138 m. The new feature layer reduces the RMSE to the longitudinal direction by matching with the measured streetlights.

## 7. Conclusions

This paper proposed a crowd-sourced mapping process of a new feature layer to reduce the costs caused by re-driving mapping vehicles and the delays of the addition of new feature layers. The process is performed in individual intelligent vehicles and the map cloud.

1. The mapping process applies the HD map-based GraphSLAM algorithm. Since the matching information between features acquired from perception sensors and features in the existing HD map is considered, the process increases the reliability and accuracy of the new feature layer. In addition, the new feature layer has high consistency with the existing HD map.

2. In order to compensate for the errors of the low-cost sensors, the map cloud integrates crowd-sourced new feature layers based on the RLS algorithm. Since the algorithm recursively estimates the integrated feature whenever each new feature layer is inputted from each intelligent vehicle, the algorithm is effective in integrating individual new feature layers into the integrated layer.

3. Simulations were performed to analyze the effects of the sensor noises caused by the low-cost sensors. Experiments in real driving environments evaluated the performance of the crowd-sourced mapping for the new feature layer. In addition, the localization performance with the new feature layer was evaluated by comparing it with the localization performance without the new feature layer.

This paper presents the crowd-sourced mapping process of the new feature layer using multiple intelligent vehicles. The approach has limitations in that the performance of the algorithm is largely affected by the performance of the landmark feature detector. In order to overcome the dependency of the detector, the authors plan to research the crowd-sourced mapping process based on the signal level features.

## Figures and Tables

**Figure 12:**
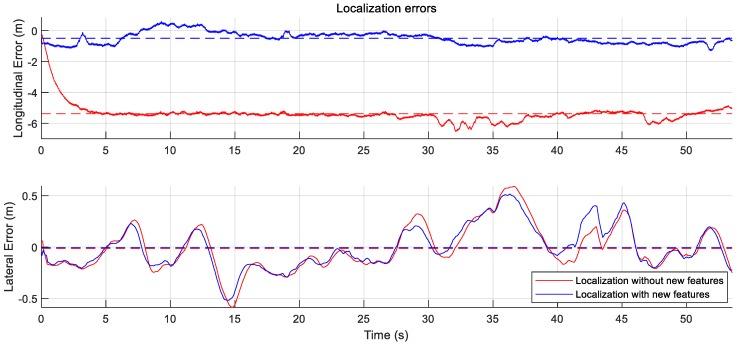
Localization errors. (top) the error to the longitudinal direction and (bottom) the error to the lateral direction. The longitudinal errors based on the localization with the new feature layer is more reduced than the errors based on the localization without the new feature layer.

**Table 1:** Sensor noise modeling.

Simulated Sensors	Sensor Noises
Distance of point feature (m)	$N(0,{\left(l_{beacon}\right)}^{2})$
Heading of point feature(deg)	$N(0,{\left(2.5l_{beacon}\right)}^{2})$
Distance of polyline feature (m)	$N(0,{\left(l_{lane}\right)}^{2})$
Heading of polyline feature (deg)	$N(0,{\left(2.5l_{lane}\right)}^{2})$

**Table 2:** Means and standard deviations of streetlight errors.

Mean (m)/Std. (m)	w/o HD Map	with HD Map	Integration
Euclidean error	5.2718/0.6877	4.5284/0.6418	0.5458/0.3543
Lateral error	0.4651/0.2506	0.1825/0.1564	0.1184/0.0939
Longitudinal error	5.2445/0.6944	4.5220/0.6418	0.5181/0.3638

**Table 3:** RMSE of localization Errors from Figure 12.

RMSE (m)	w/o New Features	With New Features
Lateral RMSE	0.2214	0.2077
Longitudinal RMSE	5.4138	0.6261
